# Preserved motion perception and the density of cortical projections to V5 in homonymous hemianopia

**DOI:** 10.1093/braincomms/fcae436

**Published:** 2024-12-09

**Authors:** Witaya Sungkarat, Thana Chaeyklinthes, Kunlawat Thadanipon, Gordon T Plant, Panitha Jindahra

**Affiliations:** Department of Radiology, Faculty of Medicine Ramathibodi Hospital, Mahidol University, Bangkok 10400, Thailand; Faculty of Health Science Technology, Chulabhorn Royal Academy, Bangkok 10210, Thailand; Advanced Diagnostic Imaging Center, Faculty of Medicine Ramathibodi Hospital, Mahidol University, Bangkok 10400, Thailand; Department of Clinical Epidemiology and Biostatistics, Faculty of Medicine Ramathibodi Hospital, Mahidol University, Bangkok 10400, Thailand; Department of Brain Repair and Rehabilitation, University College London, London WC1N 3BG, UK; Division of Neurology, Department of Medicine, Faculty of Medicine Ramathibodi Hospital, Mahidol University, Bangkok 10400, Thailand

**Keywords:** Riddoch phenomenon, visual motion perception, homonymous hemianopia, diffusion tensor imaging, neuronal plasticity

## Abstract

Following a unilateral post-chiasmal lesion of the geniculo-striate pathway, patients develop homonymous visual field defects. Using classical perimetry, patients with ‘complete’ homonymous hemianopia are unaware of stimuli in the affected hemifield. However, some show preserved vision in the affected hemifield in which the conscious perception of moving stimuli is preserved (Riddoch phenomenon). Prior evidence suggests that preservation of a direct pathway from the lateral geniculate nucleus to visual area 5 (bypassing the primary visual cortex) may be the basis of this type of residual vision. The aim of the present study was to investigate the possibility of a correlation between preserved motion perception in hemianopia and the fibre connectivity density of the underlying pathways. This research was a case-control study carried out in a tertiary care centre between 2019 and 2021. Participants (*n* = 48) were divided into two groups: patients with homonymous visual field defects (*n* = 20) and normal controls (*n* = 28). All participants underwent Humphrey field analysis (outcome = visual field index); kinetic perimetry (outcome = %correct); brain MRI; and diffusion tensor imaging probabilistic tractography (outcome = fibre connectivity density). The difference between %correct in kinetic perimetry and visual field index in Humphrey field analysis provided an indication of the level of preserved motion perception. A significant positive correlation was found between the fibre connectivity density of contralateral lateral geniculate nucleus-contralateral visual area 5 and the preserved motion perception (rho = 0.5965, *P* < 0.0012) and between the fibre connectivity density of contralateral visual area 5-contralateral lateral geniculate nucleus and the preserved motion perception (rho = 0.5635, *P* < 0.0012) after adjusting with the Bonferroni method. The area under the curve was 0.7947 for the preserved motion perception in reflecting the fibre connectivity density of contralateral lateral geniculate nucleus-contralateral visual area 5, and 0.7660 for the preserved motion perception in reflecting the fibre connectivity density of contralateral visual area 5-contralateral lateral geniculate nucleus. We have demonstrated an extensive network of pathways connecting visual areas in the two hemispheres via the splenium of the corpus callosum. To our knowledge, this is the first report of a correlation between the preserved motion perception and the fibre connectivity density of the pathways underlying the Riddoch Phenomenon (specifically bilateral lateral geniculate nuclei to visual area 5 contralateral to the lesion). The difference between %correct in kinetic perimetry and visual field index measures the preserved motion perception and is related to the underlying neural damage. The methodology has the potential to evaluate and monitor patients with hemianopia.

## Teaser

Sungkarat *et al*. present a significant advancement in understanding the preserved visual motion perception in patients with homonymous hemianopia. It is correlated with fibre connectivity density underlying the neural network through diffusion tensor imaging. The methodology provides evidence of neuronal plasticity and a novel method for monitoring these patients.

## Graphical Abstract

**Graphical Abstract:**
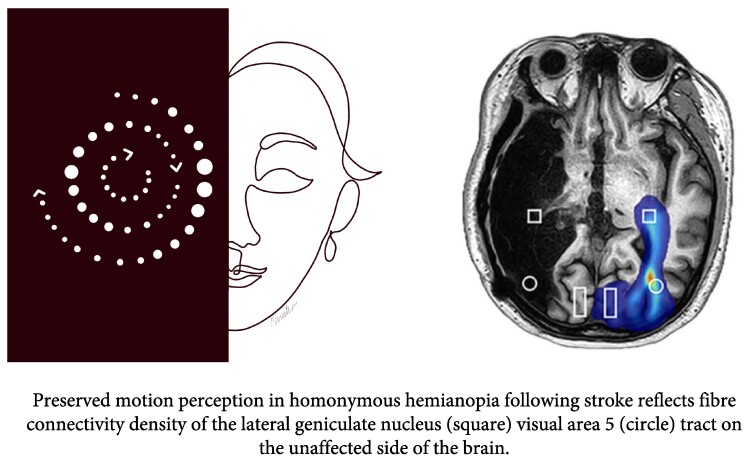

## Introduction

Following a unilateral post-chiasmal lesion of the geniculo-striate pathway, patients develop varying degrees of visual loss affecting the contralateral visual field. This is commonly homonymous hemianopia (HH) but partial preservation of the visual field is also seen (e.g. macular sparing or quadrantanopia). Bilateral occipital lobe damage can cause cortical blindness, effectively a bilateral HH. However, whatever the pattern of visual field loss, residual vision may be demonstrable within the affected area. In the past, perimetric testing was predominantly kinetic (e.g. Bjerrum Screen and Goldmann methods) but in recent years the advent of automated methodology has resulted in the use of static testing. Visual field examination by classical perimetry requires a patient response indicating conscious visual awareness. Patients with ‘complete’ HH are unaware of stimuli in the affected hemifield, indicating the fundamental role of striate cortex (V1) in conscious visual perception.^1^ Other patients, however, may show evidence of residual vision. This may be unconscious perception in regions of the visual field, which is perimetrically blind. This ability is known as ‘blindsight’ and was so called because the patient performed better than chance at detecting a visual stimulus when guessing, without conscious awareness.^2-4^ A range of evidence has suggested that sub-cortical pathways mediate blindsight, where there is a total loss not only of V1 but all other cortical visual areas following hemispherectomy.^5^ A further class of residual vision, first defined by Riddoch is preservation of conscious perception of moving but not static stimuli (known as the Riddoch phenomenon [RP] or stato-kinetic dissociation).^6^ A study of such a patient found that contrast sensitivity to temporally modulated stimuli was within normal limits: sensitivity far greater than that demonstrated in ‘classical’ blindsight.^7^ A later study demonstrated that a hemianopic patient could discriminate the direction of movement of visual stimuli in the blind hemifield but not the size or shape of the target.^8^ Recent studies have conflated the concept of blindsight with this form of residual vision by adopting a criterion of residual vision in hemianopia where there is no response to static stimuli on automated perimetry.^9^ As the patients studied demonstrated conscious detection, this finding should more properly be classified as the ‘RP’. Stato-kinetic dissociation can be demonstrated following lesions at a number of locations in the visual pathway and in at least one neurodegenerative disorder.^10-17^ It is also known that patients may exhibit both conscious and unconscious residual vision under appropriate conditions.^12^

With advances in imaging techniques such as functional magnetic resonance imaging (fMRI) and diffusion tensor imaging (DTI), the neural basis of conscious and unconscious visions can be identified.^1^ There is increasing evidence of neuronal pathways sub-serving preserved motion perception in hemianopia: namely, a direct pathway from the LGN to V5/MT+ . This pathway bypasses the geniculo-striate pathway (LGN-V1).^18-20^ Indeed, it has been suggested that if the pathway from LGN ipsilateral to the lesioned V1 is also damaged, the patient will not show RP.^9^ The aim of the present study was to investigate the possibility of a correlation between preserved motion perception in hemianopia and the fibre connectivity density (FConnD) of the underlying visual pathways described above. One previously unanswered question is the relative contributions to the incidence or RP of V5/MT+ ipsilateral and V5/MT+ contralateral to the lesion: particularly as in many cases of HH (other than pure V1 damage) the ipsilateral direct pathway from LGN to V5/MT+ will also be damaged.

## Materials and methods

### Study protocol

We conducted a case-control study at the Ramathibodi Hospital from June 2019 to June 2021. The Ethical Clearance Committee on Human Rights Related to Research Involving Human Subjects, Faculty of Medicine Ramathibodi Hospital, Mahidol University approved the study. All participants gave written informed consent to participate in the study. All participants underwent static perimetry using a Humphrey visual field analyser (HFA; HFA75oi, Carl Zeiss Meditec, Inc.), kinetic perimetry (KP), brain MRI and DTI. Demographic data were collected from electronic medical records, including sex, age, diagnosis, disease duration, laterality (left, right or bilateral damage) and visual field parameters.

### Participants

The participants were categorized into two groups: patients and controls. We recruited patients with HH and bilateral HH with macular sparing (BHH) from a stroke unit and a neurology clinic. The inclusion criteria for the patient group were: (i) age >18 years; (ii) HH or BHH confirmed with static perimetry. Reliable performance on HFA (with sufficient residual vision for maintenance of fixation); (iii) KP performed (see below); and (iv) a brain lesion located anywhere along the geniculo-striate pathway. The inclusion criteria for the controls were (i) age >18 years, (ii) normal HFA, (iii) KP performed and (iv) normal brain MRI.

### Humphrey visual field analyser

All patients underwent HFA 24-2 at the initial presentation. The HFA projects a series of static white spots of light with varying light intensities over five orders of magnitude, from 10 000 apostilbs (asb) to 0.1 asb at a number of pre-determined locations in the bowl. The stimulus size is 4 mm^2^, and the duration is 0.2 s. Testing is monocular, and appropriate lens correction is provided. Patients fixate on a central target, monocularly responding with a button press when a target is perceived. The visual field index (VFI) quantifies performance, ranging from 100% correct to the total loss at 0%. The mean VFI of the two eyes was calculated. HFA is a standard perimetric technique for measuring conscious perception of statically presented stimuli. Thus, a patient with complete HH will have a VFI of ∼50%. It should be noted that the patients were instructed to press whenever a stimulus was seen and were not encouraged to guess: the methodology will not, therefore, demonstrate blindsight. Also, the methodology tests out to 24° of visual angle from fixation and does not, therefore, test the entire visual hemifield (the normal visual field measures 90° temporally to central fixation, 50° superiorly and nasally and 60° inferiorly). Therefore, residual vision at the extreme periphery will not be detected.

### Kinetic perimetry

We employed a bespoke web-based KP method in this study, Rama Motion Perimetry (https://www.rama.mahidol.ac.th/vrt/rmp_v1/© Mahidol University. All rights reserved), in a room lighted by an electric bulb. The visual stimuli are small black dots (negative contrast, luminance 0 cd/m^2^) moving in a clockwise direction with a velocity of 6°/ms. The dots appear randomly in time and location on a computer screen (luminance 1 cd/m^2^) within the central 24° of the visual field. The viewing distance is 30 cm. Patients were instructed to fixate on a central target binocularly and to click a spacebar as soon as they perceived any movement regardless of form perception. The result is expressed as percent correct. Therefore, the difference between %correct in KP and %correct VFI in HFA (KP-VFI) provides an indication of the level of preserved motion perception or degree of stato-kinetic dissociation. KP is considered as valid when errors (false negative and false positive) detected in the programme are close to zero. The greater the difference, the greater the level of residual motion perception. Once again, as patients are not encouraged to guess, such as in a two-alternate forced choice paradigm, this residual vision should not be considered blindsight as the criterion for a response depends upon conscious perception.

### Magnetic resonance imaging

Brain MRI was performed using a 3.0 Tesla scanner (Ingenia; Philips Healthcare, Best, the Netherlands). We used standard brain MRI protocols, including axial and coronal T1-weighted images, T2-weighted images, axial fluid-attenuation inversion recovery images, diffusion-weighted images (DWI) and apparent diffusion coefficient.

### Diffusion tensor imaging

A Philips Ingenia MRI system at 3.0 Tesla (Philips Healthcare, Best, the Netherlands) software release 5.3 was used. Data acquisition was acquired with 32 diffusion directions with the following parameters: b-value = 1000 s/mm^2^, TR = 3569 ms, TE = 88 ms, matrix size = 128 × 128 × 70 and voxel size = 1.8 × 1.8 × 2.4 mm.

### Software

On a Linux computer workstation, DTI data were analysed with FMRIB Software Library (FSL) v5.0.11 (http://www.fmrib.ox.ac.uk/fsl/fdt/index.html).

### Pre-processing

Brain DWIs using a b-value of 0 s/mm² (B0) were pre-processed by the FSL brain extraction tool, resulting in brain segmentation from the skull tissue. FSL Eddy's current correction tool was utilized to reduce inconsistent image distortion. Diffusion parameters and the FSL probabilistic diffusion model were appraised at each voxel using the BEDPOSTX algorithm, creating all files necessary for running probabilistic tractography. FSL probtractx tool (a crossing fibre model included) using ¼ voxel-stepped size was applied for the probabilistic tractography. It was constrained by a 90° threshold for maximal curvature (angle difference) and the fibre orientation with the slightest angular deviation from the previous tractography step.

### Region of interests and tracking

**Figure 1:**
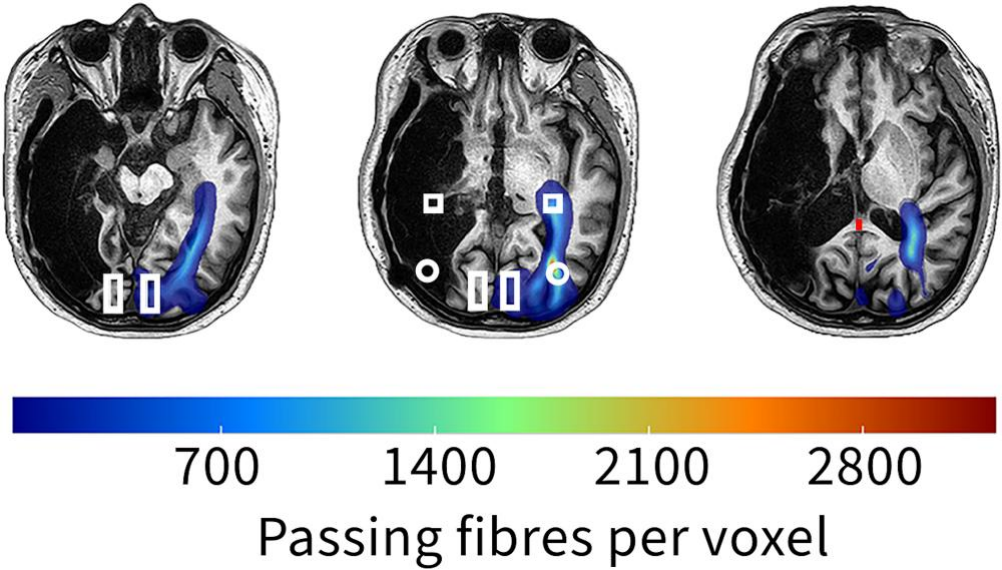
**A representative example of probabilistic tractography in a 32-year-old male with a right middle cerebral artery territory infarct.** ROIs are placed in white matter regions encompassing LGN. No tractography is found on the right tracts. Left LGN-V1 and LGN-V5 tracts appear normal (shown in blue). Shape representation: square, LGN; rectangle, V1; circle, V5; red line, corpus callosum.

**Figure 2:**
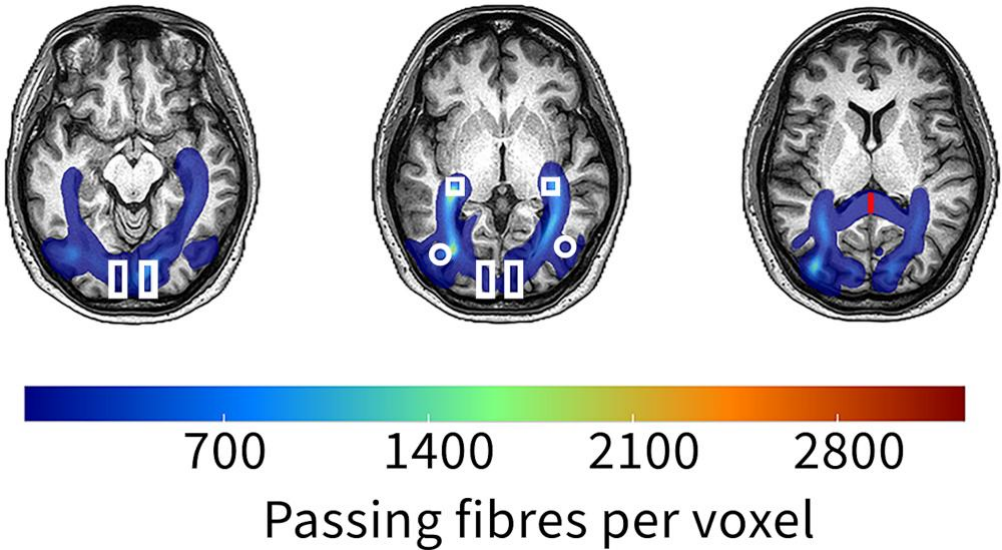
**A representative example of probabilistic tractography in a normal 26-year-old female.** ROIs are placed in white matter regions encompassing LGN-V1 and LGN-V5 tracts. Bilateral LGN-V1 and LGN-V5 tracts appear normal (shown in blue). Shape representation: square, LGN; rectangle, V1; circle, V5; red line, corpus callosum.

Probabilistic fibre tract analyses were performed in the native diffusion space between 7 region of interests (ROIs); the splenium of the corpus callosum (CCP), ipsilateral lateral geniculate nucleus (iLGN), contralateral LGN (cLGN), ipsilateral V1 (iV1), contralateral V1 (cV1), ipsilateral V5/middle temporal visual area (iV5) and contralateral V5/middle temporal visual area (cV5). The ROIs were prefixed with either ipsilateral (i) or contralateral (c) regarding their location in the brain relative to the side of the lesion. In cases of bilateral lesions, we used the ipsilateral prefix for the side with the more extensive lesion and the contralateral for a smaller one. ROIs were drawn by hand on the structural image for each participant to be within the Jülich atlas definitions of each area (as implemented in FSL). Examples can be seen in Figs. 1 and 2. There was an overlap between the lesions and ROIs in some regions. The splenium of the corpus callosum is located in the posterior part of the corpus callosum.^21^ The LGN is located in the posterior thalamus. It is surrounded anteriorly by the cerebral peduncle and posteriorly by the origin of the optic radiation.^22^ The primary visual cortex (V1) is located in and around the calcarine sulcus.^23,24^ V5 is located in the parieto-temporo-occipital cortex, mainly at the junction of the inferior temporal sulcus and the anterior limb of the inferior temporal sulcus.^21,25,26^

**Figure 3:**
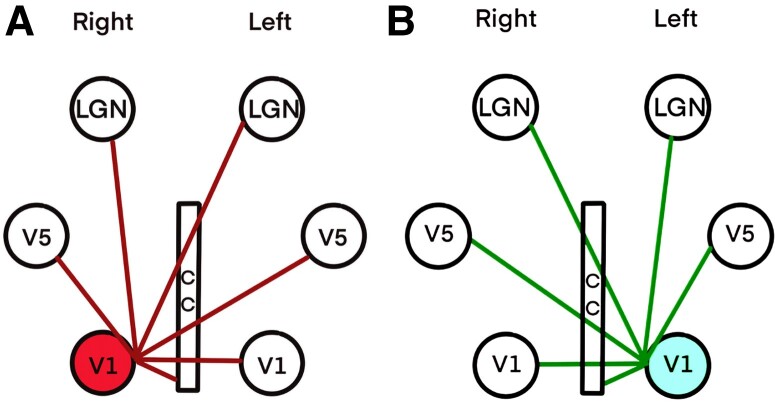
**Representative examples of seeding-target pairing used in this study:** (**A**) the seeding ROI is the right V1 (shown in a red circle) connecting to right LGN, right V5, left LGN, left V1, left V5 and corpus callosum (CC). (**B**) The seeding ROI is the left V1 (shown in a blue circle) connecting to left LGN, left V5, right LGN, right V1, right V5 and corpus callosum.

We calculated the FConnD for all 42 possible connections between these 7 ROIs. For each pair of neuronal connections, a seed mask was marked as the starting point of the tractography and the other as its destination. For example, the abbreviation iV1-cLGN stands for a connection between ipsilateral V1 and contralateral LGN (Fig. 3). The FConnD was evaluated for each paired ROI at the destination ROI. FConnD was the number of fibre paths passing from seeding ROI through target ROI divided by the volume of target ROI.

### Primary outcomes

KP-VFI and FConnD.

### Statistical analysis

Demographic characteristics were described as mean and standard deviation (SD) for normally distributed data and as median and interquartile range (IQR) for non-normally distributed data. The Student’s *t*-test was used to compare the mean between the two groups with respect to age, ROI voxels (overall, CCP, iLGN, cLGN, iV1, cV1, iV5 and cV5) and ROI volume (overall, CCP, iLGN, cLGN, iV1, cV1, iV5 and cV5). Pearson χ^2^ test was used to compare the proportions of sexes between the two groups. Median regression was used to compare the KP-VFI between the two groups. Student’s *t*-tests were used to compare the mean ROI voxels and volume between the two groups. To take account of the variability in the number of voxels among the participants, the DerSimonian-Laird random-effects meta-analysis model was used to pool the FConnD within each of the case and control groups. Then, the pooled means of these values were compared between the two groups with the Student’s *t*-tests. Spearman correlation coefficients were calculated to determine correlations between FConnD and KP-VFI. For those with statistically significant correlations, we further estimated the Spearman correlation coefficients between the FConnD and disease duration, between FConnD and age, between KP-VFI and disease duration, and between KP-VFI and age. In addition, a receiver operating characteristic analysis was carried out for KP-VFI. The area under the curve (AUC) was used to determine the ability of KP-VFI to reflect the underlying FConnD. The FConnD cut-off score at the 25th percentile was used as a reference. Sub-group analysis was performed according to lesion location. A Mann–Whitney U-test was conducted to compare the KP-VFI between V1 and the radiation lesions. Bonferroni correction was applied to correct for 42 multiple tests with the threshold for statistical significance at <0.0012 after correction, respectively. All statistical tests were performed using Stata17 (StataCorp, 2021, College Station, TX, USA). Since no preliminary work was done, we utilized a consecutive sample of all eligible patients for the study.

## Results

Forty-eight participants were enrolled in the study and divided into two groups: patients (*n* = 20, 8 females, median KP-VFI = 10.2, median disease duration = 224 days) and normal controls (*n* = 28, 19 females, median KP-VFI = 0). There were no significant differences in age, sex, KP-VFI, ROI voxels (overall, CCP, iLGN, cLGN, iV1, cV1, iV5 and cV5) and ROI volume (overall, CCP, iLGN, cLGN, iV1, cV1, iV5 and cV5) between the two groups (Table 1). In the patient group, there were nine cases of unilateral posterior cerebral artery territory infarction (PCAI); five unilateral middle cerebral artery territory infarction (MCAI); two unilateral temporal haemorrhage; one unilateral parietal-temporal craniotomy for epilepsy treatment; one posterior cortical atrophy; one PCAI and MCAI; and one left temporal haemorrhage post-craniectomy with right PCAI.

**Table 1:** Characteristics between groups

Characteristics	Patient	Controls	*P*-value
	*N* = 20	*N* = 28	
Age, year, mean (SD)	54.2 (16.8)	46.6 (12.8)	0.082
Sex, *n* (%)			
Female	8 (40.0)	19 (67.9)	0.055
Male	12 (60.0)	9 (32.1)	
MP-VFI, median (IQR)	10.2 (−7.2, 27.3)	0.0 (−2.0, 0.0)	0.101
ROI volume CCP	116.4 (16.3)	121.2 (5.1)	0.150
ROI volume iLGN	324.9 (31.6)	338.6 (41.7)	0.220
ROI volume cLGN	324.9 (31.6)	338.6 (41.7)	0.220
ROI volume iV1	4079.6 (337.7)	3890.5 (497.5)	0.150
ROI volume cV1	4020.8 (504.5)	3893.8 (502.0)	0.390
ROI volume iV5	532.0 (280.0)	615.6 (95.3)	0.150
ROI volume cV5	597.2 (310.6)	590.8 (23.7)	0.910
Disease duration (days), median (IQR)	224 (526.5)	NA	NA
Bilaterality, *n* (%)			
0	0 (0%)	28 (100%)	NA
1	17 (85%)	NA	NA
2	3 (5%)	NA	NA
Left brain lesion, *n* (%)	9 (45%)	NA	NA
Right brain lesion, *n* (%)	8 (40%)	NA	NA

Pearson χ^2^ test was used to compare the proportions of sexes between the two groups. Median regression was used to compare the KP-VFI between the two groups. Student’s *t*-tests were used to compare the mean ROI voxels and volume between groups.

### Fibre connectivity density comparisons between the two groups

The mean values of CCP-iV1, CCP-cV1, iLGN-iV1, iLGN-iV5, cLGN-iV1, iV1-CCP, iV1-iLGN, iV1-cLGN, iV1-cV1, iV1-cV5, cV1-CCP, cV1-iV1, iV5-CCP, iV5-iLGN, iV5-cLGN, iV5-iV1, iV5-cV1, iV5-cV5, cV5-CCP and cV5-iV1 in the patient group were lower than those in the controls. Significant differences were observed in CCP-cV1, iLGN-iV5, cLGN-iV1, iV1-CCP, iV1-iLGN, iV1-cLGN, iV1-cV1, iV1-cV5, cV1-CCP, cV1-iV1, iV5-iLGN, iV5-cLGN, iV5-iV1 and cV5-iV1. When the mean of the patient group was higher than that of the controls, there were significant differences in CCP-iLGN, iLGN-CCP, iLGN-cV1, cLGN-iV5, cLGN-cV5 and iV1-iV5 (Supplementary Table S1).

### Correlations between fibre connectivity density and KP-VFI

**Figure 4:**
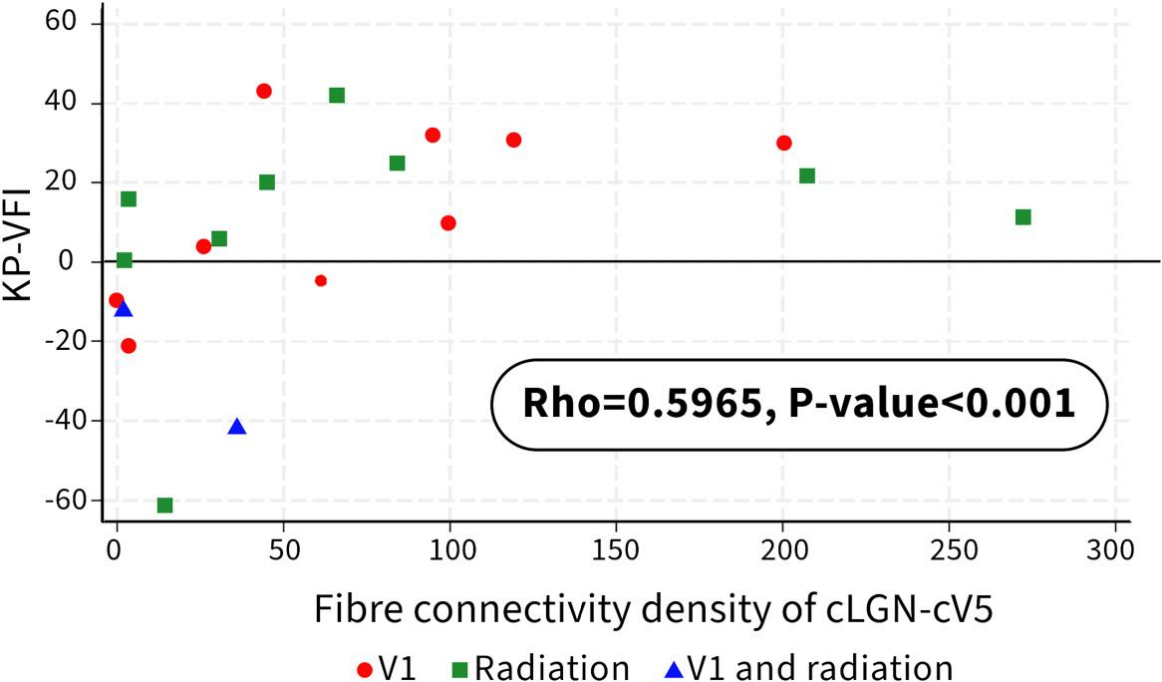
**A scatter diagram showing a relationship between KP-VFI (preserved motion perception) and FConnD of cLGN-cV5.** Significant correlations exist between the FConnD of cLGN-cV5 and KP-VFI (Spearman correlation: r = 0.5965, *P* < 0.0012).

**Figure 5:**
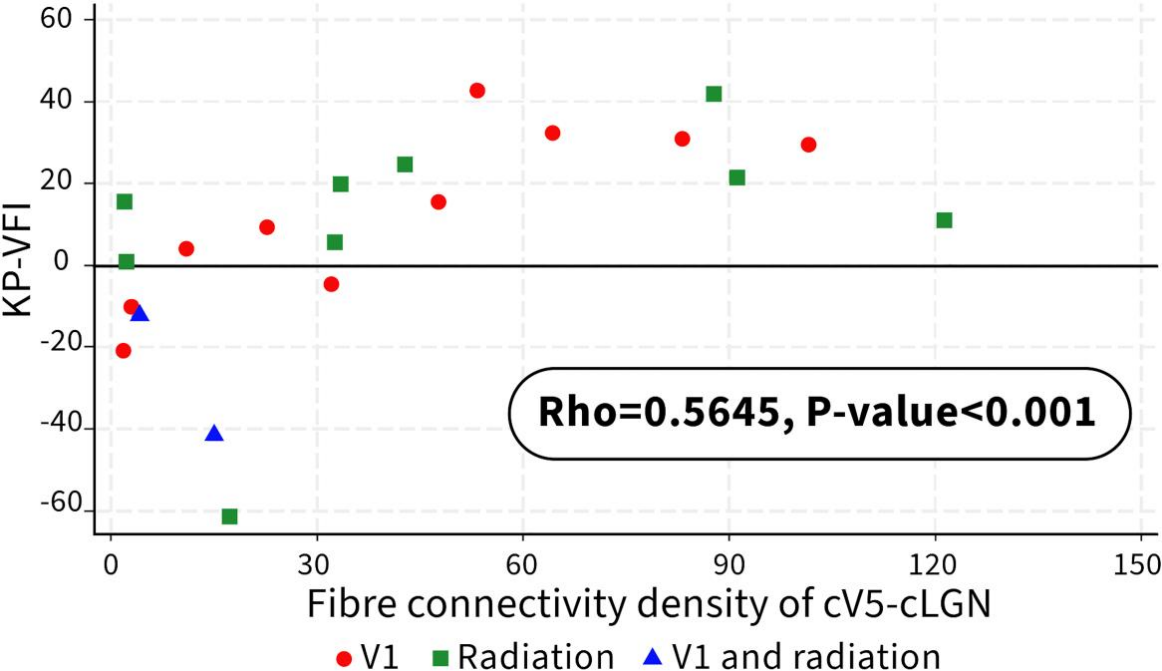
**A scatter diagram showing a relationship between KP-VFI (preserved motion perception) and FConnD of cV5-cLGN.** Significant correlations exist between the FConnD of cV5-cLGN and KP-VFI (Spearman correlation: r = 0.5635, *P* < 0.0012).

Significant correlations existed between the FConnD of cLGN-cV5 (Spearman correlation: r = 0.5965, *P* < 0.0012) and KP-VFI, and between the FConnD of cV5-cLGN and KP-VFI (Spearman correlation: r = 0.5635, *P* < 0.0012; Table 2, Figs. 4 and 5).

**Table 2:** The spearman correlations between the FConnD and MP-VFI with a significant level of <0.0012 after the Bonferroni correction

FConnD	Rho	*P*-value	FConnD	Rho	*P*-value
CCP-iLGN	0.1787	0.267	cV1-CCP	0.0764	0.630
CCP-cLGN	−0.1404	0.374	cV1-iLGN	0.2555	0.102
CCP-iV1	−0.3958	0.010	cV1-cLGN	0.3601	0.020
CCP-cV1	−0.0724	0.648	cV1-iV1	−0.1729	0.272
CCP-iV5	−0.0063	0.968	cV1-iV5	0.0613	0.699
CCP-cV5	0.3199	0.039	cV1-cV5	0.3404	0.028
iLGN-CCP	0.2953	0.058	iV5-CCP	−0.0429	0.787
iLGN-cLGN	0.2389	0.127	iV5-iLGN	−0.0078	0.961
iLGN-iV1	−0.0616	0.697	iV5-cLGN	−0.0405	0.798
iLGN-cV1	0.0807	0.610	iV5-iV1	0.0227	0.886
iLGN-iV5	0.1048	0.507	iV5-cV1	−0.1038	0.512
iLGN-cV5	0.4130	0.007	iV5-cV5	0.2595	0.097
cLGN-CCP	−0.1578	0.317	cV5-CCP	0.3288	0.034
cLGN-iLGN	0.1262	0.424	cV5-iLGN	0.4300	0.005
cLGN-iV1	−0.4515	0.003	cV5-cLGN	0.5635	<0.0012
cLGN-cV1	0.2197	0.162	cV5-iV1	−0.0233	0.883
cLGN-iV5	−0.1646	0.296	cV5-cV1	0.1855	0.239
cLGN-cV5	0.5965	<0.0012	cV5-iV5	0.1727	0.273
iV1-CCP	−0.249	0.112			
iV1-iLGN	−0.1408	0.372			
iV1-cLGN	−0.2363	0.132			
iV1-cV1	−0.2318	0.139			
iV1-iV5	0.0857	0.588			
iV1-cV5	0.1436	0.363			

### Correlations between disease duration and other parameters

There was no significant correlation between disease duration and KP-VFI (r = 0.42, *P* = 0.06), between disease duration and the FConnD of cLGN-cV5 (r = 0.24, *P* = 010) and between disease duration and the FConnD of cV5-cLGN (r = 0.17, *P* = 0.24) after adjusting with Bonferroni correction.

### Correlations between age and other parameters

There was no significant correlation between age and KP-VFI (r = −0.07, *P* = 0.65), between age and the FConnD of cLGN-cV5 (r = 0.22, *P* = 0.12) and between age and the FConnD of cV5-cLGN (r = 0.03, *P* = 0.86) after adjusting with Bonferroni correction.

### Receiver operating characteristic analysis

The AUC was 0.7947 (95% CI 0.6323–0.9571) and 0.7660 (95% CI 0.5752–0.9569) for KP-VFI in reflecting the FConnD of cLGN-cV5 and cV5-cLG, respectively (Supplementary Figs. S1 and S2). The FConnD cut-off point score at the 25th percentile was used as a reference.

### Sub-group analysis

We further divided the patients into three sub-groups regarding lesion locations, namely, V1 (*n* = 9), optic radiation (*n* = 9) and both lesions (*n* = 2) (Tables 3 and 4). The Mann–Whitney U-test revealed that the difference between V1 and radiation with respect to KP-VFI was not statistically significant (*P* = 1).

**Table 3:** Sub-group clinical characteristics

Lesion location	V1	(*n* = 9)	Radiation (*n* = 9)		V1 and radiation (*n* = 2)	
	Median	IQR	Median	IQR	Median	IQR
Age	59	9	52	34	64.5	21.5
Duration (days)	244	405	480	1490	126.5	16.5
KP (Median, IQR)	61.77	70.59	73.53	80.9	4	NA
(Mean, SD)	56.20	24.97	66.33	25.06	4	2.55
VFI (Median, IQR)	48.5	58	53.5	65	30.75	NA
(Mean, SD)	43.61	21.62	57.50	7.22	30.75	18.74
KP-VFI (Median, IQR)	9.44	30.6	15.9	21.53	−26.75	NA
(Mean, SD)	12.59	22.27	8.83	29	−26.75	21.28

KP, kinetic perimetry; VFI, visual field index; KP-VFI, kinetic perimetry-visual field index; *n*, number of participants in each sub-group; IQR, interquartile range; SD, standard deviation; NA, not available.

**Table 4:** Sub-group FConnD

Lesion location	V1	(*n* = 9)	Radiation (*n* = 9)		V1 and radiation (*n* = 2)	
	Median	IQR	Median	IQR	Median	IQR
CCP-iLGN	56.29	94.69	55.19	66.52	31.92	NA
CCP-cLGN	64.41	77.63	84.96	87.79	56.71	NA
CCP-iV1	63.66	76.81	36.47	59.24	62.23	NA
CCP-cV1	68.10	115.69	67.83	94.69	90.47	NA
CCP-iV5	2.73	4.59	23.72	47.60	34.23	NA
CCP-cV5	14.52	31.17	11.32	12.98	12.17	NA
iLGN-CCP	80.83	158.82	47.45	91.02	62.84	NA
iLGN-cLGN	25.78	91.20	27.14	42.92	28.56	NA
iLGN-iV1	33.68	110.70	22.69	40.26	47.31	NA
iLGN-cV1	8.83	20.30	5.53	7.11	28.29	NA
iLGN-iV5	10.21	31.93	12.40	43.39	6.18	NA
iLGN-cV5	5.73	10.65	1.38	4.50	13.94	NA
cLGN-CCP	62.90	92.51	63.80	95.97	104.86	NA
cLGN-cLGN	31.78	45.90	13.22	26.77	37.71	NA
cLGN-iV1	4.07	7.36	7.15	10.96	7.64	NA
cLGN-cV1	114.10	184.84	129.43	169.48	57.53	NA
cLGN-iV5	1.43	1.83	2.47	14.18	14.01	NA
cLGN-cV5	61.21	99.18	44.79	83.97	18.99	NA
iV1-CCP	157.70	645.46	216.46	368.05	142.75	NA
iV1-iLGN	25.38	55.44	37.80	52.56	30.31	NA
iV1-cLGN	13.41	15.04	27.14	74.91	25.51	NA
iV1-cV1	218.52	536.52	300.55	454.89	87.14	NA
iV1-iV5	21.63	87.37	56.65	132.44	163.53	NA
iV1-cV5	2.08	11.50	5.46	30.40	2.22	NA
cV1-CCP	288.85	332.31	283.99	507.60	503.56	NA
cV1-iLGN	16.18	37.64	9.41	27.63	8.49	NA
cV1-cLGN	125.74	213.70	188.58	245.66	84.32	NA
cV1-iV1	223.74	373.11	196.73	262.77	172.46	NA
cV1-iV5	2.53	8.64	35.16	151.27	83.21	NA
cV1-cV5	35.48	120.38	29.25	139.80	18.06	NA
iV5-CCP	9.25	14.96	16.91	73.70	21.63	NA
iV5-iLGN	5.00	12.56	18.34	51.14	2.89	NA
iV5-cLGN	2.44	4.33	8.80	32.29	7.52	NA
iV5-iV1	42.72	50.26	30.23	44.47	130.86	NA
iV5-cV1	2.48	13.44	8.64	14.47	13.09	NA
iV5-cV5	1.68	9.19	2.39	4.75	4.31	NA
cV5-CCP	17.00	28.66	13.41	25.08	4.96	NA
cV5-iLGN	3.58	13.97	2.45	4.31	2.19	NA
cV5-cLGN	31.95	64.37	33.33	87.73	9.61	NA
cV5-iV1	1.67	7.07	3.35	7.35	1.45	NA
cV5-cV1	20.82	49.95	30.94	56.74	12.85	NA
cV5-iV5	1.18	4.75	2.65	6.29	1.99	NA

*n*, number of participants in each sub-group; IQR, interquartile range; SD, standard deviation; NA, not available.

## Discussion

Here, we show an extensive network of visual pathways connecting the LGN to both V1 and V5/MT+ and connecting the two hemispheres via the splenium of the corpus callosum. By using DTI, we measured the FConnD of every possible connection from these 7 ROIS. The FConnD of ipsilateral V1-related pathways (lesioned side of the brain) was significantly lower in the patient group than in the controls. The findings indicate a degeneration process following chronic V1 or optic radiation lesions. These results are supported by other studies.^27-31^ Meanwhile, the FConnD on the contralateral side (unaffected side of the brain) and the splenium of the corpus callosum-related connections were increased, indicating a process of regeneration. MD and FA results were compatible with those of FConnD.

The difference between KP and VFI (KP-VFI) provides a metric for the degree of preserved motion perception in the patients. The difference is around zero in controls but may be positive to varying degrees in hemianopia. No case showed the reverse: greater impairment of motion perception, emphasizing the rarity of selective damage to V5/MT+ .^13,16^ KP-VFI or preserved motion perception had a strong positive correlation with the FConnD of cLGN-cV5 and cLGN-cV5. Since disease duration and age are potential confounding factors, we calculated correlations between these factors and the FConnD without any statistical significance found. Moreover, KP-VFI measured in clinics appears to reflect the FConnD of the two neuronal connections (cLGN-cV5 and cLGN-cV5) in patients with HH. KP-VFI may be useful for monitoring preserved motion perception after spontaneous recovery or visual rehabilitation. These findings and the increase in the FConnD of the cLGN-cV5 suggest that the contralateral LGN and V5 tracts play an important role in neuronal plasticity for visual motion perception. Note that the ipsilateral LGN and V1 showed degeneration/damage with consequent impairment of function. Likewise, a previous study demonstrated that the direct LGN projections to the extra-striate cortex are responsible for rapid detection of motion stimuli in normal vision and also preserved motion perception following damage to the striate cortex.^29^ The underlying neuronal connections that facilitate preserved motion perception in hemianopia lies between the LGN and the motion area V5.^9^ V1 is bypassed. A DTI and functional MRI study revealed connections between V5/MT+ and the posterior thalamus and/or superior colliculus in normal subjects.^26^

These findings emphasize the importance of contralateral hemisphere and interhemispheric connections in the residual vision following occipital injuries. A previous hemispherectomy study supports the evidence of neuronal plasticity in the visual pathway, and the role of contralesional hemisphere activity and transcallosal interaction in functional recovery.^32^ The callosal pathway has a major function as the anatomical substrate of information processing on either side of the vertical meridian, but also has the potential to mediate the transfer of information from the entire visual field to one hemisphere. The results are consistent with previous studies.^33-35^ The visual callosal pathway relays signals from both eyes via both ipsilateral and contralateral visual pathways. It receives signals directly from the ipsilateral LGN. However, callosal activation alone is insufficient to drive V1 responses. Lesions in the splenium of the corpus callosum are known to cause higher brain dysfunctions, such as metamorphopsia, visual hallucinations, agraphia and apraxia.^33,34^ Additionally, callosal alterations occur in cases of visual loss following damage to the eyes or visual cortex, suggesting a role in neuronal plasticity.^36^

Of particular importance is the finding that the cLGN-cV5 and cV5-cLGN tracts on the unaffected side of the brain are significantly correlated with RP in the blind hemifield on the affected side of the brain. It does not follow the rule of crossing and non-crossing fibres in the classic visual pathway. This finding suggests that preservation of visual motion perception can occur as a result of activity separate from the classical retina-LGN-striate pathway and is evidence for a degree or bilateral representation of the visual field. An alternative hypothesis would be that aberrant reinnervation is accountable for the residual function.

## Conclusion

Using DTI, we have demonstrated an extensive network of pathways connecting visual areas in the two hemispheres via the splenium of the corpus callosum, confirming previous studies. Our investigation has also shown that the degree of preserved motion perception shows a positive correlation with the FConnD of the projections of contralateral V5/MT+ to the LGN bilaterally. It is important to note that our novel metric for quantifying preserved motion perception will also identify patients with preserved perception of static stimuli but with a disproportionate fall in sensitivity compared to kinetic stimuli. This group will not have been included in previous studies, which may explain why we find no correlation with projections to the V5/MT+ ipsilateral to the lesion, which has been a dominant explanation for the RP in previous studies. We presume that in the present series, there has been direct damage or retrograde degeneration of that pathway.

KP-VFI measures the relative degree of preservation of motion perception and bears a relationship to the underlying neuronal damage, as such it has the potential to evaluate and monitor patients with hemianopia. The study has also further expanded the spectrum of the RP to include lesser degrees of preservation of motion sensitivity. Finally, it should be noted that the simple detection of a moving target is not evidence of intact visual motion processing, which would require studies involving discrimination of direction and velocity, and studies of motion coherence.

## Supplementary Material

fcae436_Supplementary_Data

## Data Availability

The data supporting the findings in this study are available in this article in the supplementary section. The anonymized MRI data will be provided upon reasonable request.
