# Supplementary material for: Preserved motion perception and the density of cortical projections to V5 in homonymous hemianopia
Source: Brain Commun. 2024 Dec 9;6(6):fcae436. doi: 10.1093/braincomms/fcae436 (PMC11656197; doi:10.1093/braincomms/fcae436)
Supplement: fcae436_Supplementary_Data [file fcae436_supplementary_data.pdf]

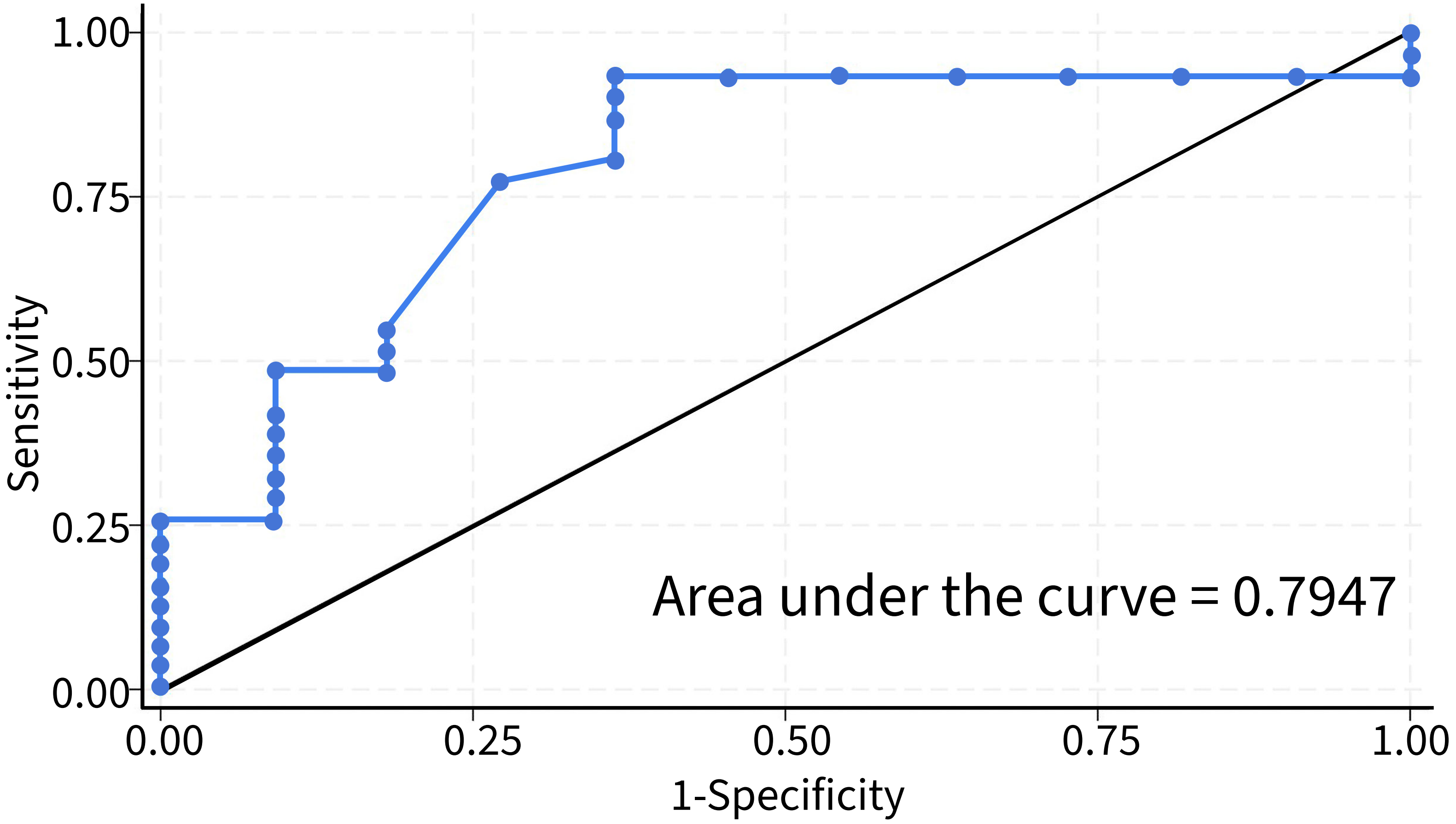

Supplementary Figure 1 Receiver operating characteristic analysis of preserved motion perception, reflecting the fibre connectivity density of contralateral lateral geniculate nucleus-contralateral V5.

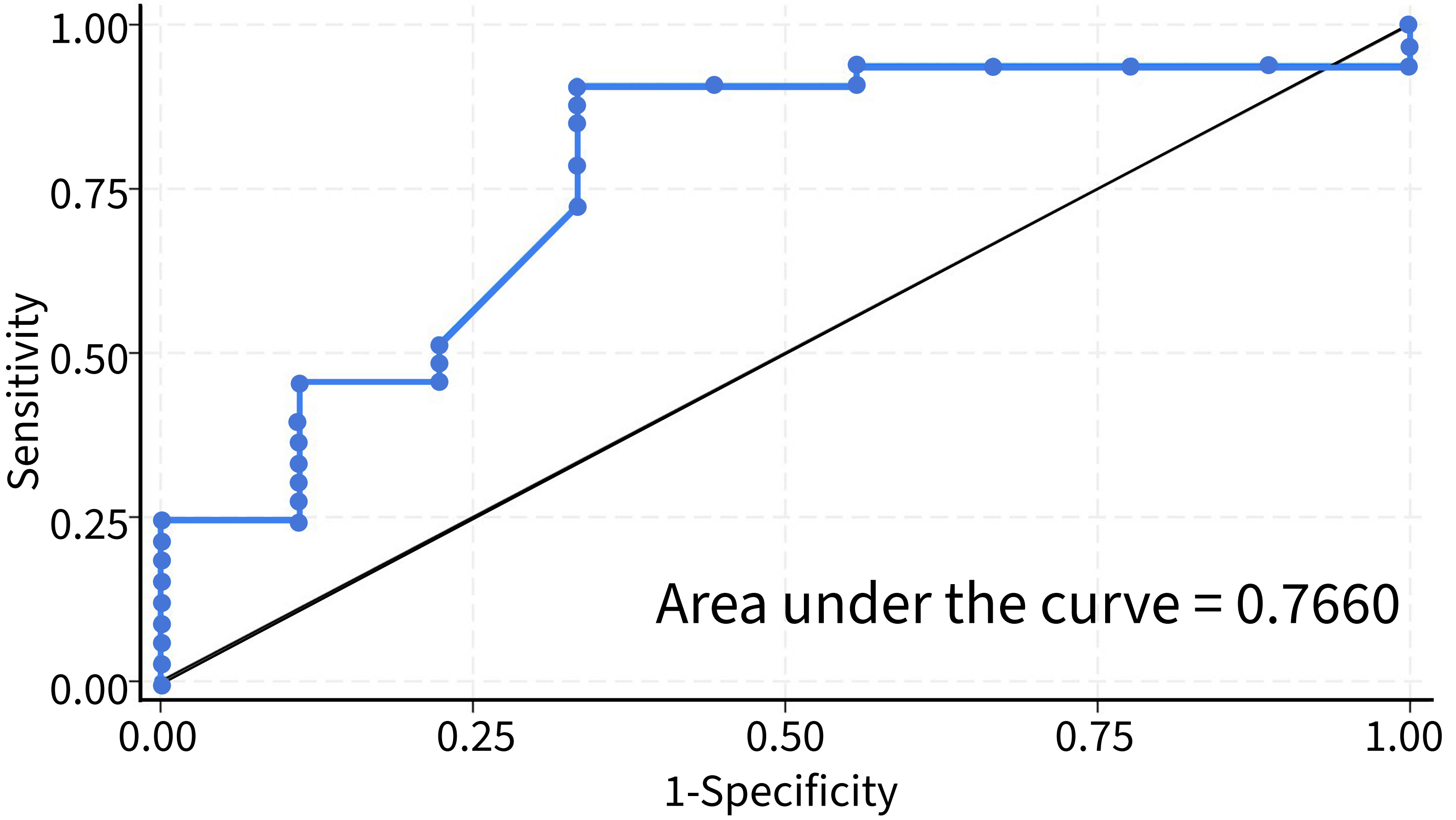

Supplementary Figure 2 Receiver operating characteristic analysis of preserved motion perception, reflecting the fibre connectivity density of contralateral V5-contralateral lateral geniculate nucleus.

Supplementary Table 1 The fibre connectivity density (FConnD) between groups. Green highlight represented that the mean FConnD of the patient group was significantly greater than that in the controls. Pink highlight represented that the mean FConnD of the patient group was significantly lower than that in the controls. The DerSimonian-Laird random-effects meta-analysis model was used with a significant level of <0.0012 after the Bonferroni correction.

FConnD	Patient			Controls			P-value
	Mean	95% CI		Mean	95% CI		
CCP-iLGN	56.477	38.867	74.087	40.905	33.09	48.719	<0.0012
CCP-cLGN	65.334	49.392	81.276	61.125	53.903	68.346	0.294
CCP-iV1	48.371	33.796	62.945	79.665	66.77	92.559	0.043
CCP-cV1	69.926	52.237	87.615	81.137	63.488	98.786	<0.0012
CCP-iV5	25.629	5.479	45.779	12.765	5.927	19.602	<0.0012
CCP-cV5	17.587	5.467	29.707	9.400	5.352	13.448	0.001
iLGN-CCP	84.104	55.205	113.004	39.438	28.852	50.023	<0.0012
iLGN-cLGN	51.552	25.662	77.441	25.427	18.043	32.812	0.650
iLGN-iV1	63.968	26.100	101.837	78.110	58.934	97.286	0.033
iLGN-cV1	14.349	5.751	22.947	9.024	6.084	11.964	<0.0012
iLGN-iV5	24.901	6.191	43.611	70.069	43.706	96.432	0.066
iLGN-cV5	10.040	3.312	16.768	4.171	-0.013	8.355	0.815
cLGN-CCP	72.692	52.458	92.927	67.488	55.628	79.349	0.406
cLGN-iLGN	42.460	18.416	66.504	26.529	20.402	32.655	0.171
cLGN-iV1	8.957	3.404	14.510	15.235	11.074	19.395	<0.0012
cLGN-cV1	140.972	98.211	183.732	110.271	78.883	141.660	0.139
cLGN-iV5	9.159	1.748	16.571	5.145	2.112	8.179	<0.0012
cLGN-cV5	79.506	37.434	121.578	30.519	19.691	41.346	<0.0012
iV1-CCP	253.081	135.366	370.796	454.969	350.465	559.473	<0.0012
iV1-iLGN	52.955	25.956	79.954	109.497	79.834	139.159	<0.0012
iV1-cLGN	28.146	15.918	40.373	51.549	35.080	68.019	<0.0012
iV1-cV1	274.871	159.508	390.233	589.600	445.965	733.235	<0.0012
iV1-iV5	73.421	34.780	112.062	45.110	19.255	70.966	<0.0012
iV1-cV5	23.917	-0.988	48.821	31.858	9.799	53.916	<0.0012
cV1-CCP	368.257	256.104	480.409	427.471	317.335	537.606	<0.0012
cV1-iLGN	20.969	11.251	30.687	19.766	15.383	24.149	<0.0012
cV1-cLGN	183.246	133.152	233.339	107.131	81.259	133.003	0.124
cV1-iV1	218.740	136.029	301.452	464.951	361.499	568.403	<0.0012
cV1-iV5	55.820	16.900	94.740	27.081	12.726	41.436	0.136
cV1-cV5	66.682	21.92	111.444	35.720	20.941	50.499	<0.0012
iV5-CCP	30.326	9.116	51.536	55.356	21.716	88.996	0.007

iV5-iLGN	19.516	2.743	36.288	62.679	39.073	86.284	<0.0012
iV5-cLGN	12.034	3.827	20.240	13.449	8.354	18.543	<0.0012
iV5-iV1	42.529	16.215	68.843	32.441	17.935	46.947	<0.0012
iV5-cV1	21.852	-0.335	44.039	43.381	7.566	79.195	0.130
iV5-cV5	2.919	1.309	4.529	4.744	0.934	8.554	0.539
cV5-CCP	26.737	11.749	41.725	28.048	14.308	41.788	0.007
cV5-iLGN	8.974	0.977	16.972	4.590	2.799	6.382	0.048
cV5-cLGN	46.947	26.546	67.348	30.377	21.593	39.160	0.001
cV5-iV1	9.656	0.877	18.436	29.015	9.871	48.159	<0.0012
cV5-cV1	38.203	14.533	61.872	29.049	22.397	35.701	<0.0012
cV5-iV5	3.201	1.099	5.304	2.814	1.213	4.414	0.596
